# Vascular Accesses for Haemodialysis in the Upper Arm Cause Greater Reduction in the Carotid-Brachial Stiffness than Those in the Forearm: Study of Gender Differences

**DOI:** 10.1155/2012/598512

**Published:** 2012-04-08

**Authors:** Daniel Bia, Edmundo I. Cabrera-Fischer, Yanina Zócalo, Cintia Galli, Sebastián Graf, Rodolfo Valtuille, Héctor Pérez-Cámpos, María Saldías, Inés Álvarez, Ricardo L. Armentano

**Affiliations:** ^1^Physiology Department, School of Medicine, CUiiDARTE, The University of the Republic, General Flores 2125, Montevideo, Uruguay; ^2^Favaloro University, Buenos Aires, Argentina; ^3^National Council of Technical and Scientific Research (CONICET), Argentina; ^4^Technological National University, Buenos Aires, Argentina; ^5^FME-Burzaco, Buenos Aires, Argentina; ^6^National Institute of Donation and Transplants (INDT), MSP School of Medicine, The University of the Republic, Montevideo, Uruguay

## Abstract

*Purpose*. To evaluate in chronically haemodialysed patients (CHPs), if: (1) the vascular access (VA) position (upper arm or forearm) is associated with differential changes in upper limb arterial stiffness; (2) differences in arterial stiffness exist between genders associated with the VA; (3) the vascular substitute (VS) of choice, in biomechanical terms, depends on the previous VA location and CHP gender. *Methods*. 38 CHPs (18 males; VA in upper arm: 18) were studied. Left and right carotid-brachial pulse wave velocity (PWV_c-b_) was measured. In *in vitro* studies, PWV was obtained in ePTFE prostheses and in several arterial and venous homografts obtained from donors. The biomechanical mismatch (BM) between CHP native vessel (NV) and VS was calculated. *Results/Conclusions*. PWV_c-b_ in upper limbs with VA was lower than in the intact contralateral limbs (*P* < 0.05), and differences were higher (*P* < 0.05) when the VA was performed in the upper arm. Differences between PWV_c-b_ in upper limbs with VA (in the upper arm) with respect to intact upper limbs were higher (*P* < 0.05) in males. Independently of the region in which the VA was performed, the homograft that ensured the minimal BM was the brachial artery. The BM was highly dependent on gender and the location in the upper limb in which the VA was performed.

## 1. Introduction

We have recently demonstrated that, in chronically haemodialysed patients (CHP), the vascular accesses determine significant changes in the upper limb native arteries' stiffness [1]. More precisely, arterial stiffness values measured in the arterial pathway of the upper limb where the vascular access was constructed were lower than those measured in its contralateral limb with intact arteries (i.e., without vascular access). Changes in arterial diameter and blood flow associated with the vascular access construction and maturation have been pointed out as factors related with arterial stiffness variations in the upper limbs where a vascular access was performed [1]. Then, theoretically, there could be differences in the carotid-brachial pathway stiffness between patients with arteriovenous fistulae (AVF) in the upper arm and those with vascular access in the forearm; with high stiffness reduction when the AVF is constructed in the upper arm. In addition, taking into account the gender dependence of arterial stiffness variations and vascular access performance [2, 3], it would be important to explore possible vascular biomechanical differences between male and female CHP. Findings regarding gender-associated differences in arterial response to vascular access construction could contribute to understand gender differences in the AVF patency rates [2, 3].

Native vessels are the conduits of first choice in performing vascular accesses for haemodialysis, arterial reconstruction, and bypassing. However, in CHP, native vessels are often damaged or not available; therefore, the only current alternative is expanded polytetrafluoroethylene (ePTFE) prostheses. This choice is very far from the ideal solution because of their poor patency rates [4, 5]. In this context, there is great interest in developing unconventional vascular substitutes to be used employed in the construction of vascular accesses in CHP.

Intimal hyperplasia stenosis in the native vessel-vascular substitute anastomotic region is the Achilles' heel for most vascular accesses in CHP. It is determined, among other factors, by native vessel-vascular substitute biomechanical mismatch (BM) [6]. Hence, the vascular substitute should have similar mechanical properties to those of the CHP native vessels [7–9]. We have previously demonstrated that cryopreservation procedures preserve the mechanical properties of fresh human muscular and elastic arteries and veins [10–12]; therefore, fresh or cryopreserved vessels could be used as an alternative in vascular access construction, resulting in BM improvement. Furthermore, we have demonstrated that BM between an upper limb native artery and a vascular substitute can be minimized by selecting the most adequate type of vascular homograft [13]. Homografts have been used in by-pass and VA construction [14–17]; nonetheless, the selection of the most appropriate homograft capable to minimize the BM in CHP upper limbs should be evaluated, taking into account gender and previous vascular access placement.

The purposes of this work were to evaluate, in CHP

1. if vascular access position (upper arm or forearm) is associated with variations in upper limb arterial stiffness,
2. the potential gender-associated differences in arterial stiffness changes related to the vascular access position, and,
3. if the vascular substitute of choice, in terms of its BM with respect to native arteries, depends on (a) previous vascular access location and (b) CHP gender.

## 2. Methods

This study was approved by our Institutional Review Board and Ethics Committee. Patients gave written agreement for noninvasive studies and documented consent was obtained for the vascular homograft procurement from deceased human multiorgan donors according to no. 14005 and 17668 legal rules of the Uruguay.

### 2.1. Noninvasive Studies in Chronically Haemodialysed Patients

Pulse wave velocity (PWV) was measured in 38 (18 males and 20 females) ambulatory patients (age:  53 ± 17  years old) who had AVF for haemodialysis in the upper limbs, at upper arm (*n* = 18), and forearm (*n* = 20) level. The term upper limb was used to define the anatomic region between the shoulder and the wrist joints. The terms upper arm and forearm were used to define the part of the upper limb between the shoulder and the elbow joints, and between the elbow and the wrist joints, respectively. Haemodialysis was performed in the morning three times a week during 4 to 5 hours, on standard bicarbonate bath. Patients had been submitted to renal function substitutive therapy for  66 ± 56  months (Table 1).

In all cases, clinical data (heart rate, brachial blood pressure, weight, age, height, hip, and waist perimeter) were obtained by the same observer previous to the PWV measurements. Body mass index (BMI) and waist-to-hip ratio were calculated for all patients.

The carotid-femoral and right and left carotid-brachial PWV measurements were performed in a stable environment, as in previous works [1, 13]. The carotid-femoral PWV, a predictor of cardiovascular events, was measured to characterize the aortic stiffness in our patients and to compare the obtained values with those expected in healthy people [18, 19]. The carotid-brachial PWV is not a predictor of cardiovascular events, but it is a technique widely used to noninvasively characterize the upper limb arterial stiffness. PWV measurements were performed using two high-fidelity strain gauges mechanotransducers (Motorola MPX 2050, Motorola Inc., Corporate 1303 E. Algonquin Road, Schaumburg, IL 60196, USA) connected to an electronic signal amplifier device [17]. Both mechano-transducers were simultaneously positioned on the skin over the carotid-femoral or carotid-brachial arteries. Signals from the same arterial pathway were acquired and analysed using software developed in our laboratory, which calculates the time delay between instantaneous arterial pulse waves. The time delay between the femoral and carotid waveforms or between the carotid and brachial waveforms (pulse transit time) was quantified using the point of maximal upstroke during systole. The direct distance between the recording sites was used to quantify the PWV. Then, using time delay data and the distance between the sensors, the arterial pathway PWV was calculated. In all patients, according to the methodology previously described by our group, several PWV measurements were obtained from a single continuous recording that included at least ten cardiac cycles [1].

Finally, blood was drawn from each patient and routine chemical analyses were performed to quantify haematocrit, haemoglobin, serum albumin, serum creatinine, calcium, phosphate, parathyroid hormone (PTH), urea, total cholesterol, HDL and LDL cholesterol, and triglycerides (Table 1). Serum creatinine was measured by the modified kinetic Jaffe reaction and recalibrated in order to calculate the estimated glomerular filtration rate (eGFR) using the Modification of Diet in Renal Disease (MDRD) Study formula [20]:

(1) $$\begin{array}{cc} \text{eGFR}\; & =\;186\cdot {\left(\text{serum}\;\text{creatinine}\;\left[\frac{\text{mg}}{\text{dL}}\right]\right)}^{-1.154}\\ & \;\cdot {\text{age}}^{-0,203}\cdot \left(0.742\;\text{if}\;\text{female}\right). \end{array}\;$$

### 2.2. *In Vitro* Studies in Vascular Substitutes: ePTFE Prostheses and Homografts

In this study we included both biological (i.e., vascular homografts) and synthetic (i.e., ePTFE) vascular substitutes. We obtained vascular segments (5 cm in length) from 10 deceased human multiorgan donors aged  31 ± 5  years: saphenous vein, femoral (muscular homograft), carotid (elastic homograft), and brachial (transitional homograft) arteries. Vascular segments were submitted to a biomechanical *in vitro* study [10–13]. Vessel harvesting techniques were in agreement with ethical and safety issues for therapeutic use. In all cases, documented consent was obtained according to no. 14005 and no. 17668 legal rules of Uruguay. Exclusion criteria agreed with the International Atomic Energy Agency (International Standards for Tissue Banks), the American Association for Tissue Bank, and the European Association for Tissue Bank.

*In vitro* dynamic analyses were performed in homografts and in 6 ePTFE (Gore-Tex Vascular graft, W. L. Gore & Associates, Inc., Flagstaff, AZ, USA) segments (5 to 6 cm in length). All conduits were mounted in a mock circulation loop and were maintained, immersed in, and perfused with Tyrode's solution (37°C, pH = 7.4, oxygenated) in a specimen chamber. The mock circulation loop consisted of polyethylene tubing, with fluid circulation powered by a pneumatic pump (Jarvik Model 5, Kolff Medical Inc., Salt Lake City, UT, USA) that allowed pressure value and waveform adjustments. Intraluminal pressure was measured with a solid-state transducer (1200 Hz frequency response, Königsberg Instruments, Inc., Pasadena, CA, USA). The segments' external diameter was measured with ultrasonic crystals (5 MHz, 2 mm diameter) [21]. The ultrasonic signal transit time, obtained during the experiment, was converted into distance by means of a sonomicrometer (Triton Technology Inc. San Diego, CA, USA). As in previous works [10–13, 17, 21], taking into account the speed of sound in biological tissues and in experimental media used in arterial *in vitro studies*, and considering our experimental conditions (i.e., temperature, vascular smooth muscle reactivity), we assumed a sound speed of 1580 m/s [22, 23].

After being placed in the specimen chamber, all segments were allowed to equilibrate under steady state flow and pressure. The simulated haemodynamic conditions mimicked those of the CHP included in the study, ensuring isofrequency and isobaric comparisons among groups. During each experimental session, pressure and diameter signals were measured under dynamic conditions, displayed in real time, digitised every 5 ms, and stored for offline analysis. All recorded samples included at least twenty consecutive beats. At the end of each experiment, in order to calculate the conduit strain, a recording of the nonpressured midwall radius (*R*_0_) was computed as the radius at 0 mmHg [17]. Afterwards, segments were weighed and instantaneous wall thickness (*h*) was calculated as

(2) $$h=r_{e}-r_{i},$$

where *r*_*e*_ and *r*_*i*_ are the external and internal radius, respectively.

The internal radius (*r*_*i*_) was calculated as:

(3) $$r_{i}=\sqrt{r_{e}^{2}-\frac{V}{\pi \cdot L}},$$

where *L* is the *in vivo* length, and *V* is the conduit volume, calculated using the segment weight and assuming a tissue density (*ρ*) of 1.06 g·cm^−3^.

PWV was calculated using the Moens-Korteweg equation:

(4) $$\text{PWV}=\sqrt{\frac{E_{\text{INC}}\cdot h}{2\cdot R\cdot \rho }},$$

where *E*_INC_ is the incremental elastic modulus and *R* is the midwall radius [17].

In order to determine *E*_INC_, values of the conduit strain (*ε*) and circumferential stress (*σ*) were calculated as

(5) $$\varepsilon =\frac{R}{R_{0}},$$

(6) $$\sigma =\frac{2P{\left(r_{e}\cdot r_{i}\right)}^{2}}{r_{e}^{2}-r_{i}^{2}}\cdot \frac{1}{R^{2}},$$

where *R*_0_ is the non-pressured midwall radius obtained as described above and *P* is the intraluminal pressure. Finally, the stress-strain loop was constructed in all cases; this allowed us to obtain the *σ*-*ε* purely elastic relationship, using the hysteresis-elimination method. *E*_INC_ was calculated as

(7) $$E_{\text{INC}}=0.75\frac{d\sigma }{d\varepsilon },$$

where *dσ*/*dε* is the first derivative of *σ* respect to *ε* [17].

Finally, the CHP native artery-vascular substitute biomechanical mismatch (BM), expressed as a percentage, was calculated as [12, 13]

(8) $$\text{BM}=\frac{\text{PW}{\text{V}}_{\text{CHP}}-\text{PW}{\text{V}}_{\text{Vascular}\;\text{substitute}}}{\text{PW}{\text{V}}_{\text{CHP}}+\text{PW}{\text{V}}_{\text{Vascular}\;\text{substitute}}}\cdot 100.$$

The BM values range between 100 and −100. A null BM (BM = 0) represents an optimal coupling or matching (i.e., vascular substitute and native vascular pathway with identical biomechanical behaviour), while values far from 0 indicate increasing mismatch. A negative value indicates that the vascular substitute is stiffer than the native vascular pathway; a positive value indicates that the native vascular pathway is stiffer than the vascular substitute.

## 3. Statistics

Measured and calculated values were expressed as mean value ± SD. A *P* < 0.05 was considered statistically significant. Unpaired *t*-test and ANOVA plus Bonferroni tests were employed. The statistical software used was the SPSS 17.0 (Chicago, IL, USA).

## 4. Results

Both noninvasive and *in vitro* studies, performed according to the methodology described above, provided high quality biological wave recordings. No technical mistakes occurred during data acquisition. As was expected, the studied CHP showed an increased aortic stiffness level, evidenced by a higher carotid-femoral PWV than the expected for age and blood pressure-matched healthy European [24, 25] or South-American [26] subjects. There were not significant carotid-femoral PWV differences across the groups (Table 1).

Patients with the vascular access for haemodialysis in the forearm and those with the vascular access in the upper arm showed nonsignificant differences in terms of age, height, weight, and waist and hip perimeters (Table 1). Similarly, with the exception of PTH levels, there were no differences between the groups regarding biochemical, and haemodynamic parameters (Table 1). There were not significant differences in eGFR levels across the groups (Table 1).

Gender comparison of clinical characteristics, biomechanical parameters, and arterial pressure values showed only significant differences (*P* < 0.05) in terms of height, haematocrit, PTH, and haemoglobin levels (Table 1).

### 4.1. Carotid-Brachial Arterial Stiffness: Vascular Access Location and Gender Dependence

**Figure 1:**
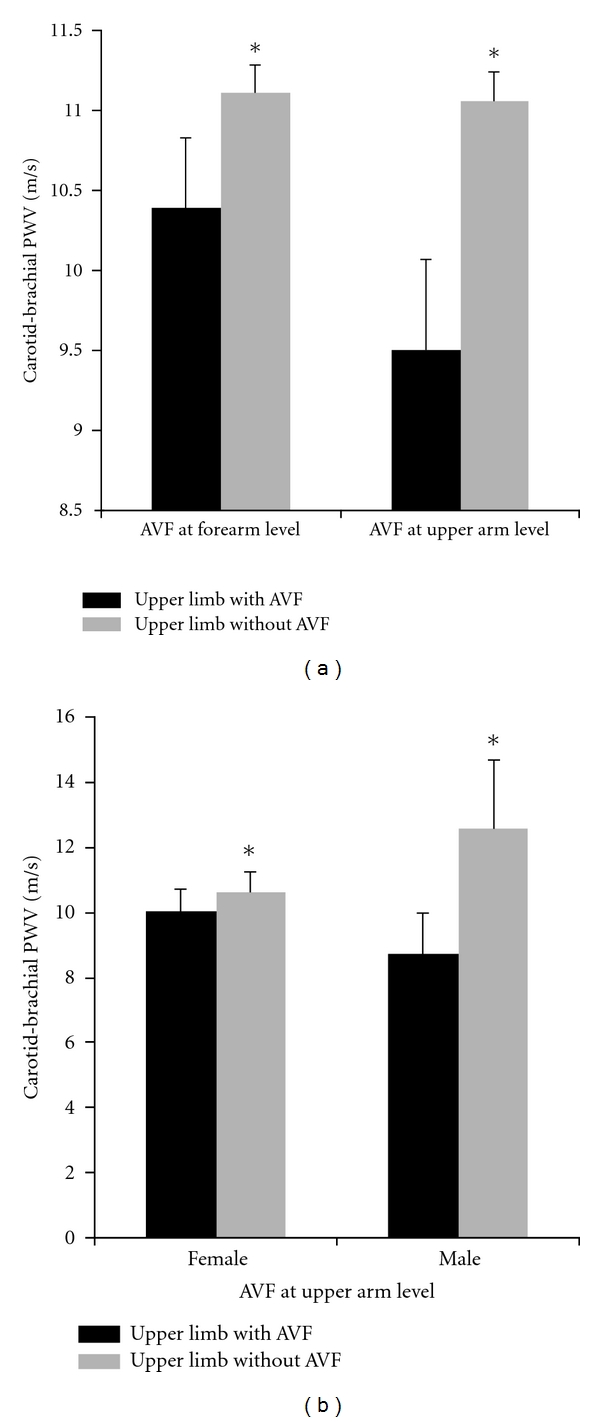
Comparison between carotid brachial pulse wave velocity (PWV) values (mean ± SD) obtained in upper limbs with arteriovenous fistulae (AVF) with respect to their contralateral intact limbs (*P < 0.05). The analysis discriminates between vascular accesses performed at upper arm and forearm level (a), and between genders when the vascular access was performed at upper arm level (b).

**Figure 2:**
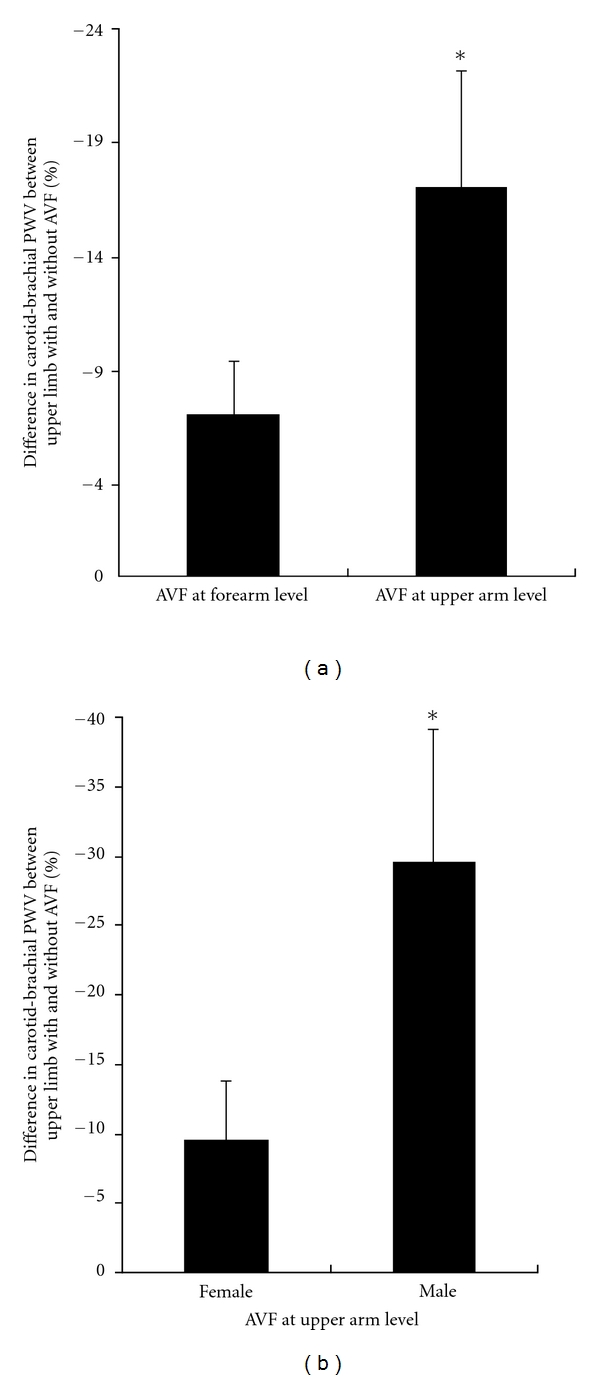
Carotid brachial pulse wave velocity (PWV) differences (mean ± SD) between upper limbs with arteriovenous fistulas (AVF) with respect to their contralateral intact limbs. Differences obtained at forearm level were lower than those obtained at upper arm level (a). At upper arm level, differences obtained in females were lower than those obtained in males (b) *P < 0.05.

Regardless of the vascular access location (upper arm or forearm), the carotid-brachial PWV was lower in the upper limb with the vascular access (*P* < 0.05) than in the contralateral one (i.e., the limb with intact arteries). When AVF was performed in the forearm, a statistically significant difference was observed between contralateral upper limbs (10.50 ± 0.47 versus 11.30 ± 0.36 m/s); a similar finding was confirmed when the AVF was constructed in the upper arm (9.60 ± 0.61 versus 11.30 ± 0.78 m/s) (Figure 1(a)). The mentioned differences were greater (*P* < 0.05) in CHP with the AVF in the upper arm than in those with the vascular access in the forearm (−17.00 ± 5.18% versus −7.10 ± 2.29%) (Figure 2(a)).

The carotid-brachial PWV measured in intact upper limbs (i.e., without AVF) of CHP described above was similar, regardless of whether the AVF was performed in the upper arm (11.30 ± 0.78 m/s) or in the forearm (11.30 ± 0.36 m/s) (Figure 1(a)).

When considering gender in the vascular stiffness analysis, it was observed that both male and female CHPs with vascular accesses in the upper arm exhibited lower PWV values (*P* < 0.05) in the limb with the AVF than in its contralateral intact limb (Figure 1(b)). More precisely, in females, PWV values obtained in the carotid brachial pathway where the AVF was performed were lower (*P* < 0.05) than in the contralateral intact limb (10.00 ± 0.67 versus 10.60 ± 0.57 m/s); similar findings were observed in males (8.70 ± 1.29 versus 12.60 ± 2.09 m/s) (Figure 1(b)). The mentioned differences were greater in males than in females (−29.60 ± 9.36% versus −9.50 ± 4.15%) (Figure 2(b)).

### 4.2. Biomechanical Mismatch between Vascular Substitutes and Native Arteries: Vascular Access Location and Gender Dependence

Human brachial artery homografts ensured the best BM values with native vessels (Table 2), regardless of gender and vascular access location. Moreover, BM values are better in females than in males with AVF performed in the upper arm. On the other hand, ePTFE prostheses showed the worst BM values.

Analysing the biological alternatives included in Table 2, we observed that the same graft can show very different values of BM. When comparing the native vessel-homograft BM values obtained in the limb with the AVF with those of the limb with intact arteries, there were differences (*P* < 0.05) regardless if the AVF was performed in the upper arm or in the forearm. Similar differences were found when comparisons were made considering gender (Table 2).

Saphenous vein and femoral and brachial artery homografts showed lower values of BM with respect to native vessels when the AVF was performed at the forearm level than those measured when the vascular access was constructed in the upper arm (*P* < 0.05), see Table 2(a). On the other hand, when using carotid artery homografts, the opposite result was obtained (*P* < 0.05) (Table 2(a)). Finally, differences in native artery-homograft BM were found between males and females, regardless of AVF location (Table 2).

## 5. Discussion

We have recently demonstrated that in CHP, arterial stiffness values measured in the arterial pathway of the upper limb where the vascular access was constructed were lower than those measured in its contralateral limb with intact arteries (i.e., without vascular access) [1]. Continuing our project, and using the same data set previously employed [1], and new original clinical and experimental data and records, in this work we analyse the carotid-brachial arterial stiffness considering the vascular access location (upper arm or forearm levels), and we analyse potential gender-related differences. The main findings were as follows.

- First, the reduction in the carotid-brachial PWV was greater in upper limbs with the vascular access located in the upper arm.
- Second, carotid-brachial PWV reduction in upper limbs with vascular access was evidenced in males and females CHP. When the AVF was constructed in the upper arm, the arterial stiffness reduction was greater in males.
- Third, independently of the AVF location (i.e., upper arm or forearm), the vascular homograft that ensured the best BM was the brachial artery.
- Fourth, the native vessel-vascular substitute BM showed significant differences, depending on the gender and the upper limb considered (intact or submitted to AVF construction).

PWV provides reliable information about arterial stiffness. In fact, at present, the carotid-femoral PWV is considered the gold standard technique to evaluate the regional aortic stiffness using non-invasive methods [24]. Furthermore, in haemodialysed patients, carotid-femoral PWV is a valuable mortality predictor [27–30]. Then, considering its clinical meaning and that PWV is a simple, non-invasive, robust and reproducible method to evaluate arterial stiffness, in this work we selected the use of PWV to determine both the aortic (carotid-femoral PWV) and the upper limb arteries (carotid-brachial PWV) stiffness. As it was expected, in CHP, carotid-femoral PWV levels were higher than the levels reported to evidence arterial subclinical alterations [24–26].

In this study serum PTH levels were elevated (as is common in CHP [31]), and differences in PTH levels were found among groups (Table 1). However, no association was found between PTH levels and carotid-femoral or carotid-brachial PWV values. Then, differences in PTH levels could not explain differences in carotid-brachial PWV reported in our work. This finding agrees with previous works, in which no association was found between serum PTH levels and aortic stiffness or risk of death or cardiovascular events [31, 32].

AVF have shown a beneficial impact on aortic stiffness; this improvement would be greater in patients with increased PWV before vascular access construction [33]. Additionally, a reduction in the carotid-brachial stiffness in upper limbs with AVF has been described [1]. In the first case, the arterial stiffness improvement has been associated to a blood pressure reduction; in the second case, it would be due to an arterial diameter increase, associated with AVF maturation. In the present study we found that the carotid-brachial PWV reduction was greater in patients with the AVF performed in the upper arm than in those with the AVF in the forearm. Therefore, we hypothesised that this reduction in arterial stiffness associated with the existence of a vascular access could result in an increase in arterial conduit and buffering function and a reduction in left ventricle afterload [24]. However, the analysis of the differences in the arterial stiffness reduction in terms of arterial function and cardiac afterload changes and their meanings was beyond this work's scope.

Stiffness differences between limbs with the AVF in the upper arm and those with the vascular access in the forearm could be related with anatomical factors since the brachial artery diameter is the highest among upper limb arteries [3]. This is an important issue since the arterial wall's intrinsic properties (i.e., the elastic modulus) and the vessel's geometrical parameters (i.e., diameter) are main determinants of the PWV (3) [24]. It is well known that PWV and arterial diameter have an inversely proportional relationship. Furthermore, the increase in diameter in vessels used in the AVF construction constitutes an important issue in the vascular access maturation process [34].

The AVF maturation and patency rates are lower in female than in male haemodialysed patients [2, 3, 34]. The origin of gender differences has not been yet elucidated. Several studies have pointed out that the lesser maturation rate in females would be due to the smaller diameter of vessels in females, but factors other than vessel geometry could also be involved in the maturation outcomes [2, 3, 34]. The differences regarding AVF evolution described above could be related to the gender dependence of age effects on arterial wall properties of upper limbs [35]. In 2000, a population study demonstrated that brachial artery diameter increase, with age in females than in males. Additionally, brachial artery compliance values did not decrease with age, on the contrary, a significant increase was observed in females, while among the male population no changes were observed [36]. According to our results, the differences in the carotid-brachial PWV between upper limbs with and without AVF were significantly higher in males than in females. This original finding was observed only when the vascular access was performed in the upper arm. The relationship between gender differences in the vascular biomechanical behaviour and in the AVF evolution should be analysed. Regarding this, it has been stated that AVF maturation needs compliant vessels that can dilate in response to blood flow increase [34]. Therefore, gender differences in vascular access construction-associated changes in arterial stiffness could contribute to the gender differences in the AVF evolution. As a consequence of the findings reported in the present study, further analyses should be performed in order to determine the clinical relevance of vascular biomechanical properties to predict the AVF outcome. More specifically, vascular stiffness analysis should be included in studies on the low performance of vascular accesses in females. Furthermore, a longitudinal study including PWV measurements before and after vascular access construction could contribute to improve the knowledge of the AVF maturation process.

Gender and location of the vascular access (i.e., upper arm or forearm) differences could be the origin of previously reported findings, in which the authors pointed out that forearm AVF have low success rate in chronically haemodialysed females [3].

When analysing the carotid-brachial PWV changes in hemodialysed subjects, at least three factors should be considered for an adequate interpretation of our results. At least in theory, the fistulas/PTFE grafts maturation could influence PWV values, since changes in AVF or grafts blood flow impedance associated with the maturation process could modify the upper limb local hemodynamic conditions. For instance, changes in local haemodynamic conditions could determine changes in the shear-stress applied to the endothelial layer, which could modify the arterial wall biomechanics through changes in the vascular smooth muscle tone. Taking into account this, looking for stable conditions, in our work the non-invasive studies were always done in mature AVF. Second and related with that stated above, it is noteworthy that our patients' average length on renal replacement therapy was 66 months. This time could differ (being higher or lower [31]) with the mean survival length on hemodialysis in other centres and/or countries. Then, the characteristics of our CHP could differ from those of other CHP groups, which should be considered at the time of extrapolating our results to other patients. Furthermore, the vascular changes associated with hemodialysis could depend on the therapy length. Then, future works should be developed to assess the potential association between the subjects' characteristics and/or the hemodialysis length and the vascular changes in CHP.

According to our results, in CHP, the location of the vascular access would result in differences in arterial stiffness evaluated through PWV measurements in the carotid-brachial pathway. In addition, as previously stated, the BM between the native vessels and the vascular substitute to be used in the vascular access construction could depend on (a) gender, (b) the evaluated limb (with or without a VA), and (c) location of the previous vascular access. Hence, the patient's characteristics and arterial biomechanical parameters should also be evaluated. Nonetheless, our results show that the brachial artery homograft would be the best vascular substitute of choice, in terms of BM improvement.

Prostheses made out of ePTFE are widely used as a second choice in peripheral vascular surgeries, among them, vascular access construction. However, it is well known that ePTFE vascular accesses have poor long-term outcomes. This could be attributed to the intimal hyperplasia development, associated, among other factors, to the native vessel-ePTFE high BM [6]. We have found that, independently of CHP gender and arterial level in which the vascular access was constructed, ePTFE prostheses always determined high BM levels. Therefore, if vascular homografts were used instead of ePTFE in the vascular access construction, the BM could be significantly diminished. Further research should be undertaken in order to evaluate the clinical meaning of the findings reported in this work.

We conclude that in CHP the carotid-brachial PWV reduction was greater in patients with the AVF performed in the upper arm than in those with the AVF in the forearm. Additionally, when the AVF was constructed at upper arm level, the arterial stiffness reduction was greater in males than in females. Finally, the native vessel-vascular substitute BM could be minimized considering: (a) the histological type of vascular homograft, (b) CHP gender, (c) vascular access location (i.e., upper arm or forearm), and (d) existence of a previous vascular access.

## Figures and Tables

**Table 1:** Clinical characteristics, biochemical and arterial parameter measurements.

	CHP AVF at the forearm (*n* = 20)	CHP AVF at the upper arm (*n* = 18)		CHP AVF at the upper arm (*n* = 18)		
				*Females *(*n* = 10)	*Males *(*n* = 8)	
	Mean ± SD	Mean ± SD	∆% with respect to AVF in the upper arm	Mean ± SD	Mean ± SD	∆% with respect to females

Age (years)	52.2 ± 4.3	51.9 ± 6.3	1	55.3 ± 7.7	45.0 ± 12.1	23
Height (m)	1.63 ± 0.03	1.69 ± 0.03	−3	1.64 ± 0.03	1.77 ± 0.04	−7*
Body weight (kg)	66.8 ± 3.4	62.9 ± 3.6	6	60.2 ± 2.5	68.3 ± 10.0	−12
BMI (kg/m^2^)	24.9 ± 1.0	22.1 ± 0.9	13	22.3 ± 1.0	21.6 ± 2.2	3
Waist circumference (cm)	91.2 ± 3.9	87.0 ± 2.1	5	87.3 ± 2.3	86.3 ± 4.9	1
Hip circumference (cm)	90.5 ± 1.7	91.4 ± 2.4	−1	90.6 ± 3.3	93.0 ± 3.2	−3
Waist/hip ratio	1.01 ± 0.04	0.96 ± 0.04	6	0.97 ± 0.05	0.93 ± 0.03	5
Hematocrit (%)	32.6 ± 1.5	33.7 ± 1.5	−3	31.9 ± 0.8	37.2 ± 3.9	−14*
Hemoglobin (g/dL)	10.3 ± 0.6	10.9 ± 0.5	−5	10.3 ± 0.4	12.1 ± 1.1	−15*
Serum albumin (g/dL)	4.0 ± 0.1	4.0 ± 0.2	−1	3.9 ± 0.1	4.3 ± 0.5	−10
Calcium (mg/dL)	9.5 ± 0.2	9.3 ± 0.2	2	9.3 ± 0.3	9.2 ± 0.1	1
Serum creatinine (mg/dL)	10.0 ± 0.8	9.7 ± 0.6	3	9.3 ± 0.6	10.6 ± 1.3	−12
eGFR (mL/min/1.73 m^2^)	5.16 ± 0.41	4.87 ± 0.25	6	4.79 ± 0,29	5.09 ± 0.66	−6
Phosphates (mg/dL)	5.0 ± 0.2	5.2 ± 0.3	−3	5.1 ± 0.4	5.5 ± 0.1	−7
Parathyroid hormone (pg/mL)	393.7 ± 75.0	232.4 ± 46.3	69**	268.2 ± 57.0	160.9 ± 75.3	67*
Serum urea (mg/dL)	152.3 ± 8.8	140.4 ± 10.1	8	132.5 ± 9.6	156.3 ± 23.7	−15
Total cholesterol (mg/dL)	175.7 ± 11.3	209.2 ± 23.7	−16	209.0 ± 36.0	209.7 ± 16.5	0
HDL cholesterol (mg/dL)	40.1 ± 3.5	41.9 ± 2.2	−4	41.2 ± 2.6	43.3 ± 4.9	−5
LDL cholesterol (mg/dL)	111.4 ± 8.5	119.6 ± 12.5	−7	124.8 ± 15.8	109.0 ± 23.1	15
Total triglycerides (mg/dL)	184.2 ± 26.8	210.8 ± 34.7	−13	196.5 ± 51.8	239.3 ± 19.2	−18
Systolic blood pressure (mmHg)	128.2 ± 5.16	133.3 ± 6.24	−4	136.7 ± 5.58	126.7 ± 16.67	8
Diastolic blood pressure (mmHg)	73.5 ± 3.53	71.1 ± 4.23	3	73.3 ± 4.94	66.7 ± 8.82	10
Heart rate (beats/min)	85.9 ± 3.35	89.9 ± 4.27	−4	88.0 ± 2.35	93.7 ± 13.45	−6
Carotid-femoral PWV (m/s)	13.5 ± 0.69	14.8 ± 1.16	−9	14.9 ± 1.58	14.4 ± 1.73	4

Mean values ± standard deviation (SD). CHP: chronically hemodialysed patients. AVF: arterio-venous fistula. BMI: body mass index. PWV: pulse wavevelocity measured in carotid-femoral pathway. Blood Urea Nitrogen (BUN, mg/dl) = urea [mg/dl] divided by 2.14. eGFR: estimated glomerular filtration rate calculated using the modification of diet in renal disease (MDRD) formula. **P < 0.05 with respect to the group with AVF at forearm level. *P < 0.05 with respect to female group with AVF at upper arm level.

**Table:** (a)

	CHP with AVF in the forearm (*n* = 20) Mean ± SD	CHP with AVF in the upper arm (*n* = 18) Mean ± SD
Femoral artery homograft		
Versus upper limb with AVF	−17.00 ± 1.87	−21.60 ± 2.38 &
Versus upper limb without AVF	−13.54 ± 1.49*	−13.79 ± 1.52*
Brachial artery homograft		
Versus upper limb with AVF	1.13 ± 0.12	−3.65 ± 0.40 &
Versus upper limb without AVF	4.67 ± 0.51*	4.41 ± 0.49*
Carotid artery homograft		
Versus upper limb with AVF	15.77 ± 1.73	11.08 ± 1.22 &
Versus upper limb without AVF	19.21 ± 2.11*	18.96 ± 2.09*
Saphenous vein homograft		
Versus upper limb with AVF	−16.63 ± 1.83	−21.24 ± 2.34 &
Versus upper limb without AVF	−13.16 ± 1.45*	−13.42 ± 1.48*
ePTFE		
Versus upper limb with AVF	−78.18 ± 8.60	−79.97 ± 8.80
Versus upper limb without AVF	−76.76 ± 8.44	−76.87 ± 8.46

**Table:** (b)

	CHP with AVF in the upper arm Females (*n* = 10) Mean ± SD	CHP with AVF in the upper arm Males (*n* = 8) Mean ± SD
Femoral artery homograft		
Versus upper limb with AVF	−19.44 ± 2.14	−26.16 ± 2.88^$^
Versus upper limb without AVF	−16.76 ± 1.84*	−8.29 ± 0.91^∗$^
Brachial artery homograft		
Versus upper limb with AVF	−1.39 ± 0.15	−8.47 ± 0.93^$^
Versus upper limb without AVF	1.38 ± 0.15*	9.95 ± 1.09^∗$^
Carotid artery homograft		
Versus upper limb with AVF	13.30 ± 1.46	6.28 ± 0.96^$^
Versus upper limb without AVF	16.01 ± 1.76*	24.27 ± 2.67^∗$^
Saphenous vein homograft		
Versus upper limb with AVF	−19.07 ± 2.10	−25.81 ± 2.84^$^
Versus upper limb without AVF	−16.39 ± 1.80*	−7.91 ± 0.87^∗$^
ePTFE		
Versus upper limb with AVF	−79.14 ± 8.71	−81.64 ± 8.98
Versus upper limb without AVF	−78.08 ± 8.59	−74.49 ± 8.19

Mean value ± standard deviation (SD) (%). CHP: chronically haemodialysed patients. The biomechanical mismatch (BM) level ranges between 100 and −100. A BM = 0 represents an optimal matching; values far from 0 indicate an increasing BM. A negative BM (from 0 to −100) indicates that the vascular substitutes were stiffer with respect to the vascular pathway; a positive BM indicates the contrary.

Statistics.

*P < 0.05 with respect to upper limb with AVF, for the same vascular substitute and group of subjects.

^&^ *P* < 0.05 with respect to CHP with AVF at the forearm, for the same vascular substitute and upper limb.

^$^ *P* < 0.05 with respect to CHP with AVF at the upper arm in females, for the same vascular substitute and upper limb.
